# Supplementary material for: Dietary Branched-Chain Amino Acids and Hyper-LDL-Cholesterolemia: A Case–Control Study Using Interpretable Machine-Learning Models in Chinese Children and Adolescents
Source: Nutrients. 2025 Oct 18;17(20):3280. doi: 10.3390/nu17203280 (PMC12567327; doi:10.3390/nu17203280)
Supplement: Supplementary file 1 [file nutrients-17-03280-s001.zip › nutrients-3888392-supplementary.pdf]

Supplementary information

Figure S1. Propensity score distribution between unmatched and matched samples 2

Figure S2. Propensity score matching QQ plot 3

Figure S3. Love plot comparing absolute standardized mean differences in propensity scores and covariates between unmatched and matched samples 4

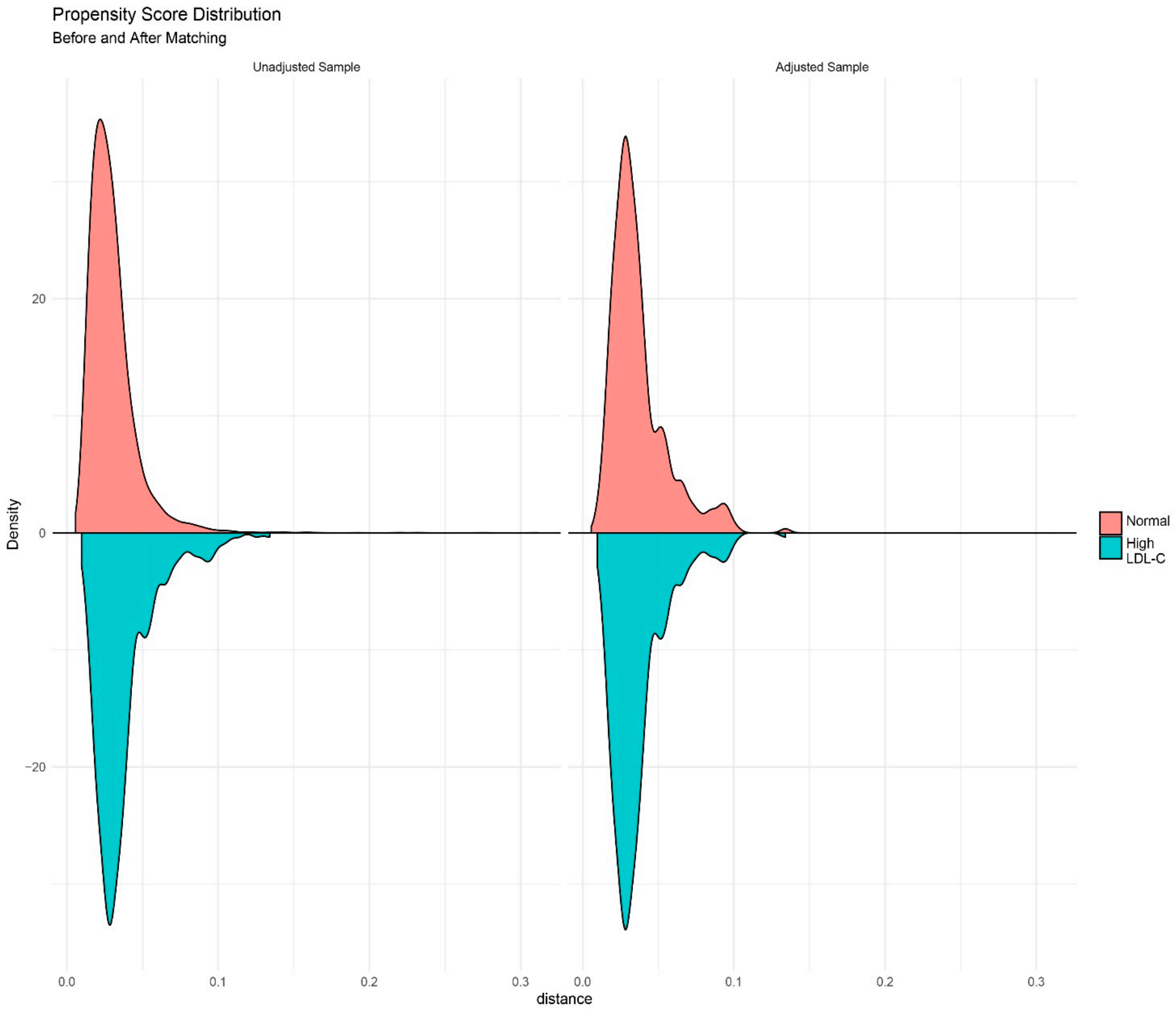

Figure S1. Propensity score distribution between unmatched and matched samples

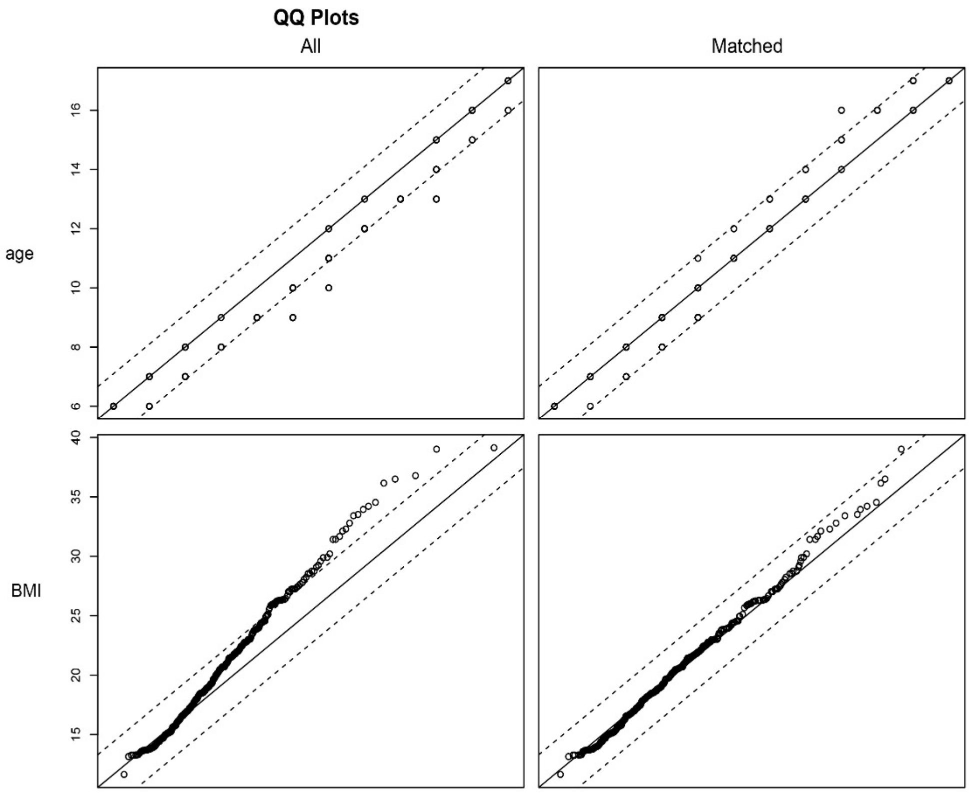

Figure S2. Propensity score matching QQ plot

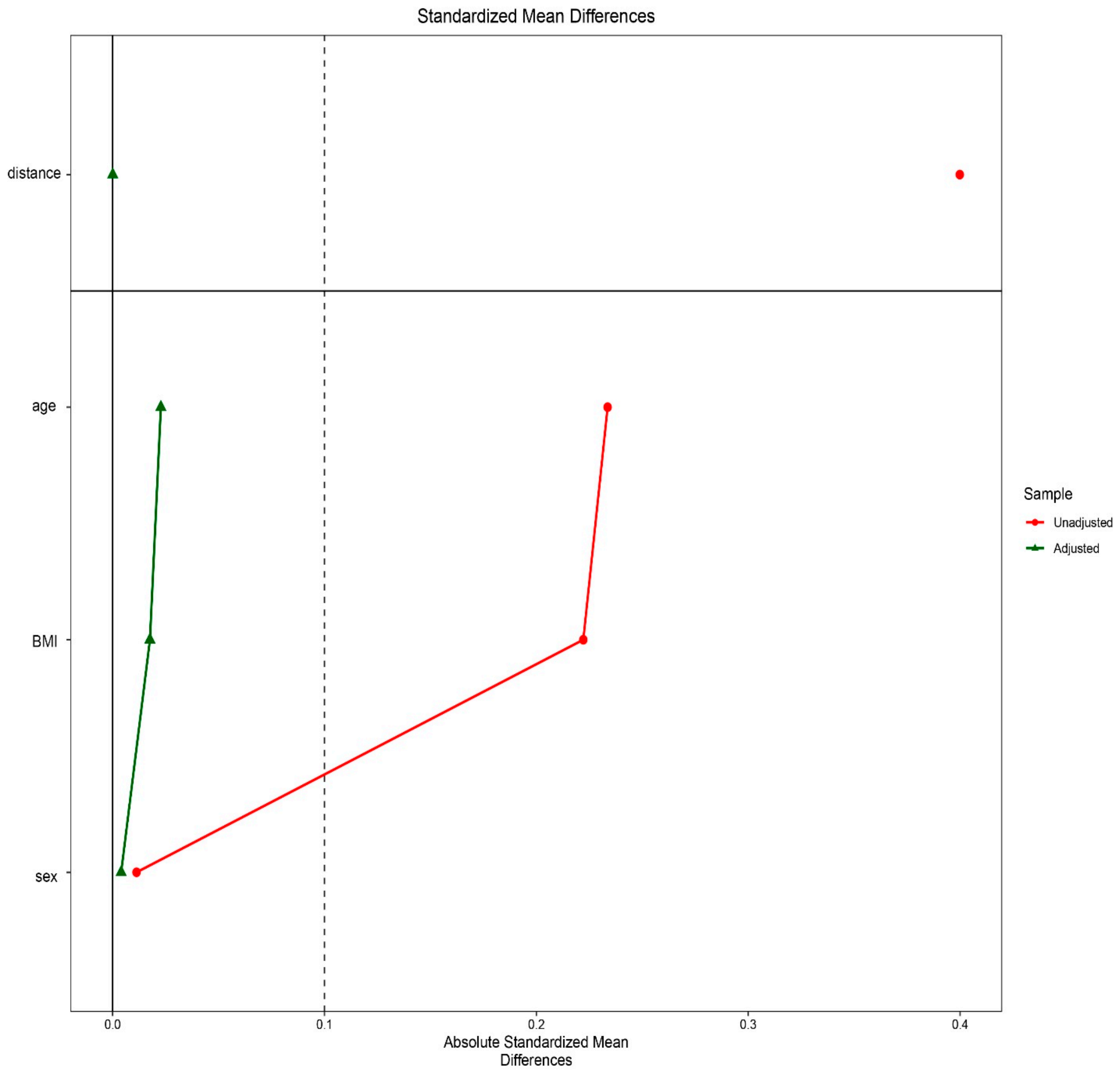

Figure S3. Love plot comparing absolute standardized mean differences in propensity scores and covariates between unmatched and matched samples
